# Diagnoses and characteristics of autism spectrum disorders in children with Prader-Willi syndrome

**DOI:** 10.1186/s11689-017-9200-2

**Published:** 2017-06-05

**Authors:** Elisabeth M. Dykens, Elizabeth Roof, Hailee Hunt-Hawkins, Nathan Dankner, Evon Batey Lee, Carolyn M. Shivers, Christopher Daniell, Soo-Jeong Kim

**Affiliations:** 1 Departments of Psychology and Human Development, Psychiatry and Pediatrics, One Magnolia Circle, Vanderbilt Kennedy Center, Vanderbilt University Medical Center, Nashville, TN 37203 USA; 2 Department of Human Development, Virginia Polytechnic Institute and State University, 366 Wallace Hall, Blacksburg, VA 24061 USA; 3 Department of Psychiatry and Behavioral Science, University of Washington, 4909 25th Ave NE, Seattle, WA 98105 USA

**Keywords:** Prader-Willi syndrome (PWS), Autism spectrum disorder (ASD), Insistence on sameness, Social impairment, Repetitive behavior, Best-estimate diagnoses, ASD screeners

## Abstract

**Background:**

A small percentage of people with autism spectrum disorders (ASD) have alterations in chromosome 15q11.2-q3, the critical region for Prader-Willi syndrome (PWS). Data are limited, however, on the rates and characteristics of ASD in PWS. Previous estimates of ASD in PWS (25 to 41%) are questionable as they are based solely on autism screeners given to parents. Inaccurate diagnoses of ASD in PWS can mislead intervention and future research.

**Methods:**

One hundred forty-six children and youth with PWS aged 4 to 21 years (*M* = 11) were assessed with the Autism Diagnostic Observation Schedule-2 (ADOS-2). An expert clinical team-made best-estimate ASD diagnoses based on ADOS-2 videotapes, calibrated severity scores, and children’s developmental histories and indices of current functioning. Children were also administered the Kaufman Brief Intelligence Test-2, and parents completed the Repetitive Behavior Scale-Revised and Vineland Adaptive Behavior Scales. Scores were compared across children with PWS + ASD versus PWS only. The performance of an ASD screener, the Social Communication Questionnaire (SCQ) and the ADOS-2 were evaluated in relation to best-estimate diagnoses.

**Results:**

Best-estimate diagnoses of ASD were made in 18 children, or 12.3% of the sample, and the majority of them had the maternal uniparental disomy (mUPD) PWS genetic subtype. Compared to the PWS-only group, children with PWS + ASD had lower verbal and composite IQ’s and adaptive daily living and socialization skills, as well as elevated stereotypies and restricted interests. Regardless of ASD status, compulsivity and insistence on sameness in routines or events were seen in 76–100% of children and were robustly correlated with lower adaptive functioning. The SCQ yielded a 29–49% chance that screen-positive cases will indeed have ASD. The ADOS-2 had higher sensitivity, specificity and predictive values. Communication problems were seen in children who were ADOS-2 positive but deemed not to have ASD by the clinical team.

**Conclusions:**

Autism screeners should not be the sole index of probable ASD in PWS; children need to be directly observed and evaluated. Compulsivity and insistence on sameness are salient in PWS and likely impede adaptive functioning. Most children with PWS only evidenced sub-threshold problems in social interactions that could signal risks for other psychopathologies.

**Electronic supplementary material:**

The online version of this article (doi:10.1186/s11689-017-9200-2) contains supplementary material, which is available to authorized users.

## Background

Prader-Willi syndrome is a neurodevelopmental disorder that results in a complex behavioral and developmental phenotype. Caused by a lack of paternally derived imprinted genes on chromosome 15q11-q13, people with Prader-Willi syndrome (PWS) typically manifest mild to moderate intellectual disability, compulsivity, rigidity, irritability, social dysfunction, growth hormone deficiencies, and hyperphagia that can lead to life-threatening obesity [1]. Most cases of PWS (65–75%) are caused by paternal deletions in the 15q11.2-q13 region and are further characterized by size. Type I deletions are approximately 500 mb larger than type II deletions. Some individuals have atypical deletions that do not encompass the breakpoints commonly seen in type I or II cases [2]. Approximately 20–30% of PWS cases are due to maternal uniparental disomy (mUPD), when both copies of chromosome 15 are maternally inherited. Occasionally, individuals have paternally inherited imprinting defects (1–3%; for a review see [3]).

Individuals with mUPD (versus deletions) are at higher risk for autism symptoms or autism spectrum disorder (ASD). They are also at heightened risk for psychotic illness, often with a depressive or affective component, which typically begins in adolescence or young adulthood [4, 5]. High risks for both disorders presumably stem from the duplication and overexpression of maternally expressed genes in the 15q11-q13 region, including UBE3A and ATP10A [6]. As well, persons with isodicentric 15 syndrome often show ASD or autism symptoms, and maternally inherited duplications of the 15q11-q13 region are relatively common findings in genetic studies of idiopathic autism, seen in 1–3% of these cases [7, 8]. Although rare, point mutations in paternally imprinted genes in the PWS region, specifically in *MAGEL2* [9] and the snoRNA region [10], were identified in a handful of children with co-occurring ASD and PWS (or strong PWS features).

Although disruptions in the PWS 15q11-q13 region are thus often implicated in ASD, data are relatively limited on rates of ASD in PWS. Studies to date are of some concern, as most have relied on autism screeners that are completed by parents, and have not directly observed or evaluated offspring with PWS. Autism screeners identify children in need of further evaluation and are not meant to be diagnostic. Even so, such tools are widely used in research to index probable ASD. Veltman et al. [11] administered the Autism Screening Questionnaire (later called the Social Communication Questionnaire (SCQ); [12]) to parents of 63 offspring with PWS aged 1 to 48 years and found that 36.5% scored above the ASQ cut-point; this rate was higher (41.4%) in participants older than 4 years of age. Those with mUPD versus deletions had higher ASQ scores. Compared to controls, Descheemaeker et al. [13] reported that 59 individuals with PWS aged 2 to 51 years had significantly elevated scores on the Pervasive Developmental Disorder Questionnaire, also completed by parents. Examining SCQ scores in 44 participants with PWS aged 3 to 37 years, Flores et al. [14] found that 35% of those with mUPD and 16.7% of deletion cases scored above the SCQ cut-point. Finally, two comprehensive literature reviews, conducted 10 years apart, found remarkably similar rates of ASD in PWS, 25.3% [15] and 26.7% [16], and higher rates of ASD were noted in mUPD cases (37.7 and 35.3%) versus those with paternal deletions (18.5 and 18.5%). No study included in these two reviews used direct observations of children to establish ASD diagnoses; most relied on screeners and a few on clinical diagnoses from unspecified sources. Beyond these concerns, the studies noted above did not address methodological challenges posed by the wide age ranges of participants, from very young children to adults.

A second complication involves the salience of repetitive behaviors in the PWS phenotype. Clinically, we find that families, educators, and other professionals often use these easily observed behaviors to raise suspicions of co-occurring ASD in PWS, even though such behaviors alone are not diagnostic of ASD. Repetitive behaviors in PWS are indeed highly reminiscent of those seen in ASD, including insistence on sameness, repetitive questioning or speech, and ordering and arranging items [17–19].

Comparing children with PWS versus ASD on the Childhood Routines Inventory [20], Graeves et al. [21] reported similarly high levels of repetitive and “just right” behaviors in both groups. In contrast, Flores et al. [14] used the Repetitive Behavior Scale-Revised (RBS-R; [22]) to compare 45, 3 to 37-year-olds with PWS to 207 children with ASD. The PWS group scored significantly lower than the ASD group, especially in the RBS-R’s restricted, ritualistic, and self-injurious behavior domains. It remains unclear, however, if these behaviors are differentially expressed in those PWS only versus PWS and co-occurring ASD.

The current study addresses these methodological concerns and also provides a more complete characterization of children with PWS and ASD compared to those with PWS only. We administered the Autism Diagnostic Observation Schedule (ADOS-2; [23]), and the Social Communication Questionnaire (SCQ; [12]) to 146 children with PWS aged 4 to 21 years. Best-estimate ASD diagnoses were made using expert clinical reviews of ADOS-2 videotapes, scores, and interview data from parents regarding their children’s current and previous educational and developmental functioning. Participants with PWS + ASD versus PWS only were subsequently compared across PWS genetic subtypes, age, gender, and their test scores from cognitive, adaptive, and repetitive behavior assessments. Finally, the study identified how well the SCQ and ADOS-2 performed in predicting ASD status as determined by the clinical review team.

## Methods

### Participants

The sample included 146 children and adolescents aged 4 to 21 years with genetically confirmed PWS. Children and families were recruited from across the country for an ongoing study on behavior and development in children and adults with PWS. Children were averaged 11.4 years of age and were evenly distributed across gender (49.3% males; 50.7% females). Regarding genetic subtypes of PWS, 52% had paternal deletions (17.8% type I deletions; 34.2% type II deletions), and 37.7% had mUPD. Fifteen children (10.3%) had either atypical deletions (*n* = 8) or imprinting defects (*n* = 7).

### Procedures

Consistent with University IRB regulations, parents of offspring with PWS provided written informed consent for the study, and children or youth with PWS provided written informed assent. Following consent and assent, a test battery was administered by trained research assistants who were highly experienced in working with individuals with PWS and their families.

#### Autism assessments

The Autism Diagnostic Observation Schedule (ADOS-2; [23]) is a widely used, standardized observational assessment for establishing autism classifications. A psychologist with ADOS-2 research training, and considerable experience in working with people with PWS, administered the ADOS-2. The ADOS-2 presents various activities aimed at eliciting social interactions and communicative and repetitive behaviors associated with autism. It was recently revised to include diagnostic algorithms and severity scores (based on raw scores) for an overall Calibrated Severity Score [24]), as well as Calibrated Severity Scores for two behavioral domains: Social Affect and Restricted and Repetitive Behaviors [25]. Calibrated Severity Scores vary by ADOS-2 Modules and child age. The revised severity scores have well-established reliability and validated cut-offs for ASD classification and allow for a standard metric across three of the four age- and language-based modules of the ADOS-2 [24].

The majority of participants in this study (*n* = 128) were administered Module 3 for verbally fluent children, while 18 were administered Module 2, designed for those who are less fluent and use phrase speech. Compared to children receiving Module 3, those administered Module 2 were younger (M’s = 11.76 versus 6.45 years, respectively; *t* (146) = −4.74, *p* < 0.001), but did not otherwise differ on cognitive or behavioral measures.

Although ADOS-2 calibrated scores are pertinent for Modules 1 to 3 in children up to age 16 years, we also administered Module 3 to youths aged 17 to 21 years (*n* = 25). We considered Module 4 (geared for adolescents and adults) and piloted it with four adults with PWS aged 24 to 30 years who were not included in the present study. These adults struggled with the more abstract items and the conversational emphasis of Module 4. ADOS-2 test guidelines note that clinicians need to determine module fit based not only on age or language skills but also on the relevance of tasks to the examinee’s interests and abilities. Instructions further note that, when in doubt, examiners should use modules that are well within reach of the examinee’s language skills. We thus opted to use Module 3 in 17–21-year-old study participants.

In order to establish the best-estimate autism diagnoses [26], the PWS research team (consisting of one PhD clinical psychologist, one MA-level clinical psychologist, BA-level research assistants, and graduate students in clinical or developmental psychology) consulted with a PhD psychologist with expertise in ASD who had long used the ADOS as a diagnostic tool in both clinical and research settings. The research team was highly experienced in PWS, while the autism expert was not otherwise involved in our PWS research program. The team met regularly to review ADOS-2 videotapes and scores and other pertinent child data collected during research visits. These other data included interviews with parents regarding their children’s developmental, medical, and educational histories; parental perspectives on their child’s current functioning; and direct assessments of children’s current cognitive, adaptive, and behavioral functioning. Cases were discussed until diagnostic consensus was achieved.

The Social Communication Questionnaire (SCQ) [12] is a 40-item parent report questionnaire that taps key symptoms of autism. Items are scored 0 or 1 (1 = the presence of the symptom), and total scores range from 0 to 39 as the first item screens for language functioning. Nineteen items rate current behavior, and 20 items apply to when the child was 4–5 years old; these are summed for a total score. The SCQ is based on the three domains included in the Autism Diagnostic Interview-Revised: communication, reciprocal social interaction, and restricted, repetitive behavior. The SCQ has discriminated between ASD and non-ASD cases with a sensitivity of 0.85 and a specificity of 0.75. We used the recommended cut off total score of ≥ 15.

#### Other assessments

A demographic questionnaire identified participant’s psychotropic medications, growth hormone treatment, age, gender, PWS genetic subtype, body mass index, family income, and parental education. These were used as correlates of ASD status and symptoms. We interviewed parents to obtain children’s developmental and medical histories, which also included a family medical history. These data assisted with the best-estimate diagnoses of ASD.

Participants were individually administered the Kaufman Brief Intelligence Test-2 (KBIT-2; [27]) which was designed for research and screening purposes and has been successfully used in previous studies of people with developmental disabilities. The KBIT-2 provides standard scores (*M* = 100, SD = 15) for a verbal, nonverbal, and overall IQ composite score. It has excellent psychometric features, including test-retest reliability of the composite (*r* = 0.90), verbal (*r* = 0.91), and nonverbal IQ scores (*r* = 0.83).

The Vineland Adaptive Behavior Scales-2 Survey Form (VABS-2; [28]) is a widely used, semi-structured interview that assesses the performance of everyday skills required for personal and social self-sufficiency in an overall composite and three domains: communication, daily living skills, and socialization. The Vineland yields domain and composite standard scores (*M* = 100; SD = 15).

The Repetitive Behavior Scale-Revised (RBS-R) assesses a wide range of restricted and repetitive behaviors in people with developmental disabilities [22]. Informants complete 43 items using a four-point Likert scale: 0 = behavior does not occur, 1 = behavior occurs and is a mild problem, 2 = behavior occurs and is a moderate problem, and 3 = behavior occurs and is a severe problem. The original RBS-2 classified items into six conceptually derived domains. However, we used Lam and Aman’s [29] five-factor solution as it was derived from factor analyses in a large cohort of people with ASD (sameness/rituals, compulsions, stereotypies, restricted interests, self-injurious behavior). Higher scores index more severe problems.

## Results

### Autism diagnoses and severity scores

#### ADOS-2 classifications and best-estimate diagnoses

Based only on ADOS-2 Calibrated Severity Scores, 32 children (21.9%) were classified as having ASD. After the clinical team reviewed ADOS-2 data and videotapes, along with children’s medical and developmental histories and current functioning, the rate was lowered to 18 children, or 12.3% of the sample. We used these second clinically informed ASD determinations in subsequent data analyses.

#### ADOS-2 positive, clinically negative children

Follow-up analyses explored possible differences between children with or without ASD, and the 14 children who were positive on the ADOS-2 yet were judged clinically to not have ASD. These 14 children did not differ from their peers in age, gender, BMI, or genetic subtype. As summarized in Additional file 1: Table S1, they also did not differ from those with PWS + ASD in their IQ and adaptive behavior standard scores. In the one exception to this pattern, the group of 14 had significantly lower scores on the Vineland Adaptive Behavior’s Communication domain (*M* = 65.80, SD = 13.87) relative to *both* the PWS-only (*M* = 78.84, SD = 14.17) and PWS + ASD groups (*M* = 75.82, SD = (11.23); *F*(2144) = 7.14, *p* < 0.001.

Based on this finding, we explored three speech-related items from participants’ developmental histories: (I) Does your child have speech/language problems? (2) If so, please describe. (3) Does your child receive speech/language therapy? Children who were ADOS-2 positive but clinically negative were more likely to have speech problems (100%) than those with PWS + ASD (64.7%) or PWS only (78.4%); *X* ^*2*^ (2) = 6.11, *p* < 0.05. Although based on parent descriptions only, no differences were found in specific problems with articulation or fluency, seen in 75 and 18.6% of the sample, respectively. However, difficulties getting thoughts into words or verbal apraxia were more frequently reported in the ADOS-2 positive, clinically negative group (76.9%) compared to those with PWS only (20.4%) or PWS + ASD (16.7%), *X* ^*2*^ (4) = 15.82, *p* < 0.01. No group differences were found in speech/language therapy, with 68% of participants receiving these services.

#### Correlates of ASD classifications

ASD diagnostic status was not significantly correlated with child age; these diagnoses were identified in 10 children and 8 adolescents. Children with versus without ASD both averaged 11 years of age (see Table 1). ASD diagnoses were not associated with the use of growth hormone treatment or psychotropic medications, body mass index (BMI), or parental education or income. ASD diagnoses were more common in boys with PWS (*n* = 13; 72.2%) than girls (*n* = 5; 27.8%); *X* ^*2*^ (1) = 4.57, *p* < 0.05). Participants with the mUPD subtype were more likely than their counterparts to receive an ASD diagnosis, *X* ^*2*^(3) = 13.31, *p* < 0.01. Of the 18 children with PWS + ASD, 14 had the mUPD subtype, 2 had imprinting defects, and 2 had type II deletions.

**Table 1:** Demographics and cognitive and adaptive functioning across PWS + ASD versus PWS-only cases

	PWS + ASD	PWS only	*T* or *X* ^*2*^
	M SD	M SD	
Age	11.77 (5.41)	11.04 (4.90)	−0.58
BMI	24.58 (9.15)	24.94 (8.47)	0.16
% Male	72.2%	48.1%	6.22**
Genetic subtypes			13.31**
Deletions	*N* = 2	*N* = 74	*N* = 76
mUPD	*N* = 14	*N* = 41	*N* = 55
Other	*N* = 2	*N* = 13	*N* = 15
KBIT-2			
Verbal IQ	64.68 (14.50)	79.92 (15.58)	2.87***
Nonverbal IQ	64.28 (20.09)	70.51 (17.22)	1.41
Composite IQ	63.78 (20.34)	72.02 (16.19)	1.95*
VABS-2			
Communication	75.82 (11.23)	77.69 (14.68)	1.27
Daily living skills	65.29 (10.48)	76.95 (17.26)	2.71***
Socialization	65.44 (11.76)	76.09 (16.84)	2.45***
Adaptive composite	64.68 (14.50)	75.33 (14.28)	2.79***

*p < .05; **p <.01; ***p < 0.001. The two PWS + ASD cases in the other PWS genetic subtype category both had imprinting defects

#### Means and correlates of ADOS-2 calibrated severity scores

Table 2 summarizes mean ADOS-2 calibrated severity scores for the overall index and the social affect and repetitive behavior domains. Not surprisingly, those with PWS + ASD versus PWS only had significantly higher calibrated severity scores.

**Table 2:** ADOS-2 calibrated severity score comparisons between children with PWS + ASD versus PWS only

ADOS-2 calibrated severity scores	PWS + ASD	PWS only	*t*, *p*
	M (SD)	M (SD)	
Social affect	8.60 (1.54)	3.11 (2.50)	4.97***
Restricted, repetitive behavior	8.81 (1.38)	4.88 (1.38)	5.86***
Overall severity	9.00 (1.41)	3.64 (2.22)	5.69***

***p < 0.001

For the sample as a whole, KBIT-2 verbal, nonverbal, and composite IQ scores were negatively correlated with ADOS-2 overall calibrated severity scores (*r*’s = −0.42, −0.33, and −0.40, respectively, *p*’s <0.001) and with social affect severity scores (*r*’s = −0.43, −0.34, and −0.41, respectively, *p*’s <0.001). IQ scores were not associated with the ADOS-2 repetitive behavior domain (*r*’s range −0.10 to −0.11). A similar pattern emerged in the Vineland’s communication, daily living skills, and socialization domains with overall severity scores (*r*’s range of −0.26 to −0.32, *p*’s <0.01) and the social affect domain (*r*’s range of −0.23 to −0.28, *p*’s <0.01), but not the repetitive behavior domain. No other significant correlations were found.

### Between-group comparisons of PWS + ASD versus PWS only

#### IQ and adaptive behavior

As summarized in Table 1, *t* tests revealed that, compared to those with PWS only, children with PWS + ASD had significantly lower KBIT-2 verbal and composite IQs and VABS daily living skills and socialization standard scores. Although both groups averaged 11 years of age, we ensured that age was not confounding results by re-analyzing data with age as a covariate. Findings remained the same.

#### ADOS-2 social and communication items

**Fig. 1:**
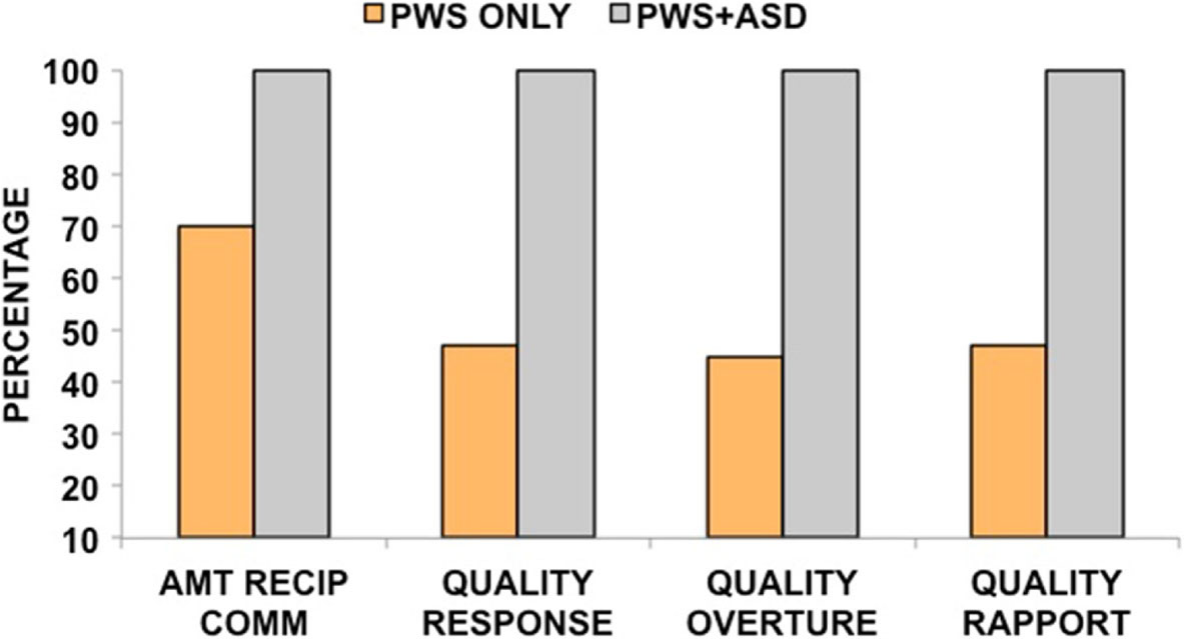
ADOS-2 Modules 2 and 3 social-communication items. Depicts the percentages of common items across Modules 2 and 3 that were scored 1 (some or possible impairment) or 2 (definite impairment). Although they did not meet ADOS-2 or clinical criteria for ASD, most (70%) of the PWS-only group still evidenced some impairment in the amount of reciprocal communication. From 45 to 47% showed abnormalities in the quality of their social overtures and responses and in their overall rapport with the examiner

**Fig. 2:**
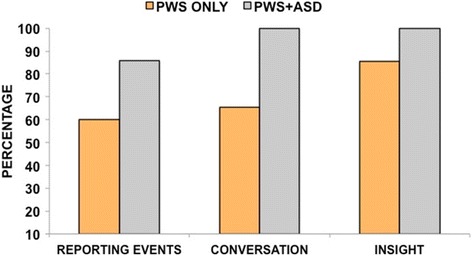
ADOS-2 Module 3 social-communication items. Shows Module 3 items that showed some degree of impairment (scored a 1 or a 2) in those with PWS only. Not shown are ADOS-2 items that were frequent and definitely present in the PWS + ASD group and infrequent or not evident in the PWS-only group

As expected, social and communicative impairments were highly prevalent in the PWS + ASD group. Because social dysfunction is often seen in people with PWS, we wondered to what extent the PWS-only group manifested problematic social and communication scores on the ADOS-2. Figure 1 shows the percentage of the PWS-only and PWS + ASD groups who received an ADOS-2 score of 1 (infrequent or possible abnormality) or 2 (definite abnormality) in four items common to both Modules 2 and 3. Despite being below the threshold for ASD, between 45 and 47% of the PWS-only group still evidenced some impairment in the amount of reciprocal communication and in the quality of their social overtures, responses, and overall rapport with the examiner. Figure 2 depicts three communication items from Module 3 for which the PWS-only group manifested some degree of impairment (a score or 1 or 2). Over half of this group had problems reporting events (60%) and conversing with the examiner (65%). Both groups had similarly high levels of poor or superficial insight, which in both groups was associated with lower composite IQ’s (PWS only *r*(126) = −0.48, *p* < 0.001; PWS + ASD *r*(14) = −0.54, *p* < 0.05). Remaining ADOS-2 items were elevated in those with PWS + ASD, but low in the PWS-only group.

#### Repetitive behavior

As shown in Table 3, *t* tests of RBS-R domains revealed that the PWS + ASD group had significantly higher scores in restricted behavior, stereotypies, and RBS-R total scores. While they also scored higher on the compulsive and sameness/rituals domains, these differences were not as robust (*p*’s <0.05). No PWS group differences emerged in the self-injurious behavior domain, which in PWS is primarily manifest as skin picking.

**Table 3:** Comparisons of mean RBS-R domain and total scores in children with PWS + ASD versus PWS only

RBS-R domains	PWS + ASD	PWS only	*t*, *p*
Sameness/rituals	17.71 (7.32)	13.85 (5.75)	−2.24*
Stereotypies	6.65 (3.71)	3.61 (3.22)	−3.44***
Self-injurious behaviors	4.23 (2.25)	5.05 (3.22)	0.504
Compulsions	13.00 (6.44)	9.12 (5.30)	−2.37*
Restricted interests	4.80 (3.27)	2.21 (2.00)	−4.13***
Total	45.71 (16.85)	33.89 (15.41)	−2.92**

*p < 0.05; **p < 0.01; ***p < 0.001

#### Correlates of repetitive behaviors

RBS-R scores were not significantly associated with age, gender, PWS genetic subtypes, and psychotropic medication or growth hormone treatment. Nonverbal and composite IQ scores were negatively correlated with the stereotypies domain (*r*’s = −0.24 and −0.22, respectively, *p*’s <0.01). Compulsivity and insistence on sameness domains were negatively correlated with the all three Vineland domains: communication (*r*’s = −0.33 and −0.55, respectively, *p*’s <0.001), daily living skills (*r*’s = −0.39 and −0.48, *p*’s <0.001), and socialization domains (*r*’s = −0.27 and −0.42, *p*’s <0.001). No other significant correlations were found.

### SCQ and ADOS-2 performances

We first determined how well the SCQ and ADOS-2 performed in relation to ASD best-estimate diagnoses made by the clinical team. We then identified the percent positive and negative agreement between these two measures, as well as the extent to which they agreed or disagreed correctly or incorrectly in relation to ASD diagnoses.

#### SCQ performance

Based on the SCQ cut-point of ≥15, 32.87% of the sample was classified as having a possible ASD. Not surprisingly, SCQ total scores were higher in those with PWS + ASD, *M* = 19.56, SD = 4.89, compared to the PWS-only group, *M* = 10.93, SD = 6.22; (*t* (143) = −5.13, *p* < 0.001.)

The test sensitivity of the SCQ, 77.78%, indicates that ASDs were accurately detected by the SCQ in the majority of participants with PWS + ASD (see Table 4). The SCQ also accurately ruled out ASD in the majority of those cases who did not have ASD, with a test specificity of 73.44%. Further, the SCQ’s negative predictive value (NPV) indicated that there is a high probability (95%) that those who screen negative for ASD will indeed not have an ASD diagnosis.

**Table 4:** SCQ and ADOS-2 performances in relation to best-estimate ASD diagnoses

	SCQ performance in relation to ASD diagnoses	ADOS-2 performance in relation to ASD diagnoses
	% 95% CI	% 95% CI
Sensitivity	77.78 (28.36–93.49)	100.00 (78.12–1.00)
Specificity	73.44 (64.91–80.85)	89.06 (82.00–93.67)
PPV	29.17 (16.95–46.06)	56.25 (37.88–73.16)
Adjusted PPV	48.71 (33.41–64.21)	75.75 (59.11–88.06)
NPV	95.92 (88.88–98.88)	100.00 (95.93–1.00)

Adjusted PPV’s used a higher estimation of ASD in PWS (~25%) based on previous literature and the formula [adjusted PPV = sensitivity × prevalence/sensitivity × prevalence + (1−specificity) × (1−prevalence)]

*CI* confidence interval, *PPV* positive predictive value, *NPV* negative predictive value

However, 28 participants were falsely classified as having a possible ASD on the SCQ when the team determined that they did not, and 5 children diagnosed with ASD were not detected on the SCQ. As such, the SCQ had a low positive predictive value (PPV) of 29.17%, suggesting a very low probability that a child with PWS who screens positive on the SCQ will indeed have an ASD.

PPV’s vary greatly depending on the overall prevalence of a disease or condition, and as such, a lower PPV and higher NPV are not unusual given the low rate of PWS + ASD (12.3%) in our sample. We thus calculated an adjusted PPV based on the higher estimates of PWS + ASD noted in the literature of ~25% [15, 16], using the formula [adjusted PPV = sensitivity × prevalence/sensitivity × prevalence + (1−specificity) × (1−prevalence)]. This more liberal calculation yielded a PPV of 48.71%.

#### ADOS-2 performance

The test sensitivity of the ADOS-2 was 1.00, indicating that it accurately detected all cases of children with PWS + ASD; none were missed. The test specificity of 89.06% suggests that the ADOS-2 also ruled out ASD in the majority of children who did not receive ASD diagnoses. Further, the NPV of 1.0 suggests a high probability that children with PWS who fall below ADOS-2 thresholds will not have an ASD diagnosis (see Table 4).

With the 14 false positive cases, however, the PPV was lower, indicating a 56.25% chance that children classified as having ASD on the ADOS-2 will indeed be diagnosed with ASD. Given the previously described interdependency between PPVs and prevalence, we also calculated the adjusted PPV for the ADOS-2. As expected, the adjusted PPV for the ADOS-2 was higher, 75.75%.

#### SCQ and ADOS-2 performance

Without a standard referent (i.e., best-estimate ASD diagnoses) sensitivity and specificity analyses are not recommended. Instead, we calculated Cohen’s kappa and the percentage of positive agreement (PPA) and percentage of negative agreement (PNA) [30]. Additional file 1: Table S2 shows the formulas and data used to calculate the PPA and PNA. The kappa, 0.32, is considered fair and just step above poor [31]. With a PPA of 58.82% (95th CI = 41.89–74.31%) and PNA of 78.86% (95th CI = 68.30–83.90%), the two measures did a better job agreeing on the absence versus the presence of possible ASD.

The PPA and NPA index agreement between measures, not if they are correct in their agreements or disagreements. As such, we compared agreements and disagreements to ASD diagnostic status. As Table 5 shows, the two measures agreed and were correct (either positively or negatively for ASD) in 67.8% cases, and they agreed and were incorrect in 4.8% of cases. When the measures disagreed, the SCQ was incorrect 22.6% of the time, and most of these were false positives. When the ADOS-2 disagreed with the SCQ, it was wrong in just 4.8% of cases.

**Table 5:** Number of agreements and disagreements between the SCQ and ADOS-2 in relation to best-estimate ASD diagnoses

SCQ	ADOS-2	*N*	ASD diagnoses	
			+	−
+	+	20	13	7
+	−	28	0	28
−	+	12	5	7
−	−	86	0	86
Total N		146	18	128

The measures agreed and were correct in 99 cases (13 + 86), and they agreed but were incorrect in 7 (7 + 0) cases. The number of disagreements when the SCQ was incorrect, 33 (28 + 5) exceeded the 7 (7 + 0) disagreements when the ADOS-2 was incorrect

## Discussion

This is the first large-scale study to identify the rates and characteristics of ASD in a large cohort of children and youth with PWS based on standardized autism assessments combined with thorough clinical reviews. Findings provide new insights into how children with PWS + ASD differ from those with PWS only in cognitive and adaptive functioning, repetitive and social behaviors, gender, and PWS genetic subtypes. Further, by determining how well the SCQ screener and ADOS-2 performed in relation to ASD diagnoses, we generate new recommendations for future research.

Using best-estimate ASD diagnoses, our 12.3% rate of ASD in 146 children with PWS falls below the 25 to 41% reported in previous studies [15, 16]. Several factors likely account for this lower rate. First, previous estimates of ASD in PWS may be inflated because studies only reported data from ASD screeners completed by parents. Although screeners are intended to identify individuals who need further evaluation, previous PWS studies did not report taking the necessary extra steps of directly evaluating those who screened positive.

Second, the clinical team may have been too conservative in making ASD diagnoses, as reflected in the 14 children who were positive on the ADOS-2 but were judged by the team to not have ASD. It is important to emphasize that the above threshold scores on the ADOS-2 are not a substitute for a diagnosis of ASD. Instead, this observational schedule provides valuable data for experienced clinicians to use in their diagnostic formulations, along with children’s developmental and medical histories and current cognitive, adaptive, and behavioral functioning [24].

The importance of this multi-modal evaluative process is highlighted by analyses of the 14 children who were ADOS-2 positive but clinically negative. Although these children had, on average, similar cognitive and adaptive behavior scores as the PWS + ASD group, they scored significantly lower than *both* the PWS-only and PWS + ASD group on the Vineland’s communication domain. Following up this finding with developmental history data, the ADOS-2-positive, clinically negative group had higher rates of speech problems than their peers, especially in verbal apraxia or difficulties getting thoughts into words. Anecdotally, the research team observed that these children typically had reciprocal social intentions, but their struggles to communicate diminished the quality of their interactions. Because we did not obtain external confirmation of parental reports of verbal apraxia, or quantify the team’s clinical impressions, these results should be interpreted cautiously. Even so, socially reciprocal intentions in children with diminished communication skills offer a reasonable explanation for why these children did not receive ASD diagnoses. Our analyses of this group also aptly demonstrate the value of thorough clinical reviews of ADOS-2 and other pertinent child data in formulating ASD diagnoses.

A final possible bias is that children’s levels of cognitive functioning may have overly influenced the team. This is particularly relevant as children with PWS + ASD versus PWS only had, on average, lower IQ and adaptive behavior standard scores. Biases in relation to IQ could go in two directions. First, it is possible that the team simply did not recognize ASD in children who were higher functioning. This possibility, however, is offset by the fact that 33% of the PWS + ASD group had KBIT-2 composite IQs of 70 or higher (*M* = 86.88, SD = 14.09, range 72–112). A second possibility is that the team was more inclined to make ASD diagnoses in lower functioning children. However, the team’s evaluation of 14 children who were ADOS-2 positive but clinically negative argues against this possibility. These 14 children had relatively low IQ’s (*M* = 59.26, SD = 16.93), and 80% of them had IQ’s <70. Despite reaching ASD threshold on the ADOS-2, this group of predominantly lower functioning children was judged *not* to have an ASD diagnosis. The team thus appeared to adequately discriminate ASD across the IQ spectrum.

Unlike their counterparts, those with PWS + ASD had elevated stereotypies and restricted interests; both are highly characteristic of ASD. Relative to those with PWS only, the PWS + ASD group had lower verbal IQs and adaptive daily living and socialization skills. For the sample as a whole, ADOS-2 overall and social affect calibrated severity scores were negatively correlated with KBIT-2 IQ scores, especially the verbal IQ. Although calibrated scores were developed to minimize the influence of cognition and other child factors, verbal IQs still account for approximately 10–11% of the variance in the overall and social affect calibrated severity scores [24, 25]. A recent meta-analysis of 12 neurodevelopmental syndromes (not including PWS) found higher rates of ASD risk and symptoms in syndromes characterized by low or variable IQs [32]. Improved understandings of cognitive differences between idiopathic and syndromic ASD will help research that frames genetic syndromes as promising alternative windows into genetic or neurobiological factors associated with ASD in general [33, 34].

Although syndromic autism may be more equitably distributed across gender [35], we found that children with PWS + ASD were more likely to be male. It is unclear if this finding is atypical or not because previous researchers have not generally reported the gender of participants with PWS who screen positive for ASD. Until future studies can clarify this gender finding, clinicians should not use gender to help rule ASD diagnoses in or out in children with PWS.

ASD’s were predominantly seen in those with the PWS mUPD subtype; they comprised 78% of the 18 children with ASD diagnoses. Overall, 25.5% of the 55 participants with mUPD were deemed to have an ASD. mUPD is thought to stem from a rescue of trisomy 15 caused by nondisjunction of maternal chromosomes and subsequent discard of the paternal chromosome 15 [6]. ASDs were also found in two of the seven children with imprinting defects. Individuals with imprinting defects have chromosome 15’s inherited from both parents, but the paternal chromosome contains a maternal imprint, resulting in loss of paternally expressed genes in the 15q11.2-q13 PWS region. As a result, imprinting defects are functionally similar to mUPD. With the addition of the two imprinting defect cases, the rate of ASD in mUPD increases slightly to 28.0%, but still falls below previous estimates of ASD in mUPD based on screeners.

Repetitive behaviors can be meaningfully subdivided into at least two broad domains; so-called lower order repetitive sensory and motor behaviors and “higher-order” insistence on sameness ([36, 37], see also [38] for evidence of a third “circumscribed interests” factor). Aside from those with PWS + ASD, stereotypies and restricted interests were neither frequent nor problematic in this cohort. Although the self-injury domain was relatively high, this was driven by a single behavior—skin picking. Stereotypical motor and senory behaviors in ASD are often negatively associated with IQ scores, while insistence on sameness is not [36]. This same pattern of results was also found in our PWS cohort.

The most frequently occurring repetitive behaviors, seen in 76–100% of participants, involved compulsivity and insistence on sameness in routines, events, timing of events, repetitive questioning, becoming upset if interrupted, hoarding, and ordering and arranging items. Although not correlated with IQ, the RBS-R sameness and compulsive domains were robustly and negatively associated with the Vineland’s communication, daily living skills, and socialization domains. Clinically, we find that these behaviors often impede optimal adaptive functioning and are among the most difficult for parents to manage. Insistence on sameness in ASDs has been associated with specific genetic alterations [39–41], including linkages to one of several GABA_A_ receptors located in the PWS 15q11.2-q13 region [42]. PWS may thus serve as a promising model for understanding insistence on sameness in ASD in general.

While the PWS-only group did not meet threshold for ASD, they still had relatively frequent problems in sustaining conversations and in the quality and amount of their social interactions (see also [43, 44]). These findings raise the intriguing possibility that deficits in social perception or cognition in most individuals with PWS do not index autism per se, but instead contribute to other emotional, behavioral, or psychiatric disorders. Indeed, both neural and genetic studies suggest similarities between PWS, ASD, schizophrenia, and psychosis [45]. Examining evoked response potential’s (ERP) to images of faces, Key and colleagues [46] found that individuals with PWS due to mUPD, but not deletions, showed a lack of visual ERP face-specific amplitude increase in N170, a robust pattern also seen in autism and schizophrenia [47]. Based on structural magnetic resonance imaging, Lukoshe et al. [48] concluded that children with PWS due to mUPD have early deviations in brain development that are reminiscent of those in ASD or schizophrenia. Finally, several genes in the PWS 15q11-q13 region are also implicated in schizophrenia or psychosis for a review see [49]. Future work is needed that identifies specific deficits in social cognition and perception in PWS, and how these might relate to psychosis or other psychiatric disorders.

The performances of the SCQ and ADOS-2 underscore the importance of combining ASD evaluation tools with thorough clinical reviews. With the SCQ’s low positive predictive value (PPV), there was just a 29% chance (49% using the adjusted PPV) that children with PWS who screen positive will indeed have ASD. Both of these PPVs fall at or below chance levels. The ADOS-2 performed much better, with enhanced specificity and sensitivity, but it too yielded a PPV just above chance (56%), which improved to 75% using the adjusted PPV. Comparing agreement across the two measures, they did a better job agreeing on the absence versus the presence of ASD (PNP = 76.8% versus PPA = 58.8%). However, we also determined if the measures agreed or disagreed correctly, i.e., in relation to ASD diagnoses. The SCQ and ADOS-2 correctly agreed on children’s ASD status 67.8% of the time, far more than they agreed but made the wrong call (4.8%). However, when the measures disagreed, the SCQ made more wrong calls (22.6%) than the ADOS-2 (4.8%), and most of these were false positives.

On the one hand, the high false positive rate of the SCQ indicates that the SCQ is performing as it was intended—to identify children in need of further evaluation. And in clinical or educational settings, a high false positive rate is often acceptable so that more children are evaluated and none are missed who could benefit from ASD interventions. Difficulties arise, however, in PWS research that only uses ASD screeners, without follow-up evaluations of screen-positive cases. Such practices risk creating a false impression that ASDs may be quite widespread in PWS.

Our findings recommend two options for future PWS research. The fact that ADOS-2 and SCQ correctly agreed for 68% of participants suggests that the two instruments performed better together than at least the SCQ did alone. If researchers want to reduce error due to false positives, they should consider using agreements between at least two standardized indices of ASD and acknowledge that subsequent rates of ASD in PWS may be still inflated. If researchers aim to precisely identity ASD in PWS, then they need to use the ADOS-2 or other direct assessments of children. Further, these observations need to be placed in the broader context of children’s development, current functioning, and phenotypic features. Indeed, Hepburn and Moody [50] convincingly argue that children with genetic, neurodevelopmental disorders must be evaluated for ASD in the context of their syndromic phenotype and developmental stage. In PWS, for example, it is unclear what role the syndrome’s characteristic infantile hypotonia, failure to thrive and growth hormone deficiencies might play in the expression of early indicators of ASD in joint attention, shared affect, imitation, and social attention, gestures, or responses [51]. It is also unclear how such social deficits respond to growth hormone replacement therapy. Beyond expected improvements in linear height, body composition, and muscular strength, growth hormone therapy also boosts cognitive and adaptive skills in children with PWS [52].

This study had several strengths, including a large, well-characterized sample and standardized, multi-modal ASD assessments that were reviewed by experts in ASD and PWS. Even so, several shortcomings need discussion. First, the study did not include a separate measure of language function and speech/language data relied solely on developmental history interviews with parents. Direct testing of language function would have been particularly helpful in characterizing the 14 ADOS-2-positive but clinically negative children. Most children with PWS have language delay, oral motor difficulties, poor articulation, slow rate of speech, flat intonation, and language skills that may fall below their level of cognitive functioning [53, 54]. Although under some debate, recent work finds that children with ASD do not typically manifest characteristics of childhood apraxia of speech [55]. Studies have yet to determine how or if motor speech difficulties in children with PWS map onto apraxia or are better described by other language disorders.

Another limitation is that the study excluded adults with PWS. Given the scarcity of research on co-occurring ASD in adults with genetic neurodevelopmental syndromes, we opted to focus initially on children and youth with PWS. Recently, however, Sappok at al. [56] evaluated ADOS-2 performance in 79 adults with moderate to severe intellectual disabilities, with or without clinical diagnoses of ASD. They suggest modifications of some test stimuli to reflect more adult interests and found that ADOS-2 overall calibrated severity scores performed best in differentiating ASD from others. Further, de Bilt et al. [57] published revised severity algorithms for Module 4 of the ADOS that discriminated between high-functioning adults with ASD, schizophrenia, sociopathy, and controls. Collectively, this work bodes well for future studies that evaluate ADOS-2 performance in adults with PWS.

A final issue relates to the analyses of SCQ performance. Other researchers have used receiver operator curves to identify SCQ cut-points that provide optimal sensitivity or specificity for their study samples. We, however, used the recommended cut-point for this measure to enable comparisons to existing literature that used this same cut-point. We also reasoned that future PWS research would not necessarily benefit from a continued reliance on screeners, even with a revised PWS-derived cut-point.

## Conclusions

We recommend that multi-modal approaches and direct observations of children be used in future studies of ASD in PWS. Additional work is needed on gender differences in PWS + ASD, and the interplay between verbal apraxia or other language disorders and ASD diagnoses. Although individuals with the mUPD versus deletion subtype are at higher risk for ASD, it is unclear how ASD symptoms change over time, or if they relate to high risks for psychosis also seen mUPD cases. Regardless of ASD status, compulsive behaviors and insistence on sameness are salient in PWS and likely impede optimal adaptive functioning. In contrast, persistent stereotypies are unusual in PWS and when present in children may signal the need for further evaluation of their social communicative functioning and ASD status. Although social impairments fell below ASD thresholds in the majority of our sample, 45 to 70% with PWS only still evidenced some degree of impairment in the quality and amount of their reciprocal social interactions. These social impairments may be associated with other psychiatric, emotional, or behavioral disorders or simply be intrinsic to the PWS phenotype.

## Additional file

Additional file 1: Table S1. Demographics and mean cognitive and adaptive scores in PWS + ASD, PWS-only, and ADOS-2-positive, clinically negative groups. Table S2. *N*’s and formulas used to calculate percent positive agreement (PPA) and percent negative agreement (PNA) between the SCQ and ADOS-2. (DOCX 125 kb)

## Abbreviations

ADOS-2: Autism Diagnostic Observation Schedule
ASD: Autism spectrum disorder
KBIT-2: Kaufman Brief Intelligence Test
mUPD: Maternal uniparental disomy
PWS: Prader-Willi syndrome
RBS-R: Repetitive Behavior Scale-Revised
SCQ: Social Communication Questionnaire
VABS: Vineland Adaptive Behavior Scales
