# Supplementary material for: Diagnoses and characteristics of autism spectrum disorders in children with Prader-Willi syndrome
Source: J Neurodev Disord. 2017 Jun 5;9:18. doi: 10.1186/s11689-017-9200-2 (PMC5458479; doi:10.1186/s11689-017-9200-2)
Supplement: Additional file 1: Table S1. — Demographics and mean cognitive and adaptive scores in PWS + ASD, PWS-only, and ADOS-2-positive, clinically negative groups. Table S2. N’s and formulas used to calculate percent positive agreement (PPA) and percent negative agreement (PNA) between the SCQ and ADOS-2. (DOCX 125 kb) [file 11689_2017_9200_MOESM1_ESM.docx]

**Additional Table 1.** Demographics and mean cognitive and adaptive scores in PWS+ASD, PWS only, and ADOS-2 positive, clinically negative groups.

|  | **PWS + ASD** | **PWS Only** | **ADOS Yes**  **Clinical No** |  |
| --- | --- | --- | --- | --- |
|  | **M SD** | **M SD** | **M SD** | **F or *X^2^*** |
| Age | 11.77 (5.41) | 10.76 (4.89) | 12.50 (4.30) | 1.52 |
| BMI | 25.05 (8.95) | 24.83 (8.46) | 24.49 (9.25) | .016 |
| % Male | 72.2% | 46.5% | 40% | 4.65 |
| **Genetic Subtypes** |  |  |  | 18.37* |
| Deletions | N=2 | N=67 | N=7 | N=76 |
| mUPD | N=14 | N=34 | N=7 | N=55 |
| Other | N=2 | N=13 | N=0 | N=15 |
| **KBIT-2** |  |  |  |  |
| Verbal IQ | 64.68 (14.50) | 81.32 (14.37) | 68.53 (19.62) | 8.65*** |
| Nonverbal IQ | 64.28 (20.09) | 72.32 (16.58) | 59.00 (15.34) | 6.05** |
| Composite IQ | 63.78 (20.34) | 73.71 (15.22) | 59.26 (16.93) | 7.52*** |
| **VABS-2** |  |  |  |  |
| Communication | 75.82 (11.23) | 78.84 (14.17) | 65.80 (13.87) | 7.14*** |
| Daily Living Skills | 65.29 (10.48) | 78.11 (16.69) | 68.14 (17.14) | 6.14** |
| Socialization | 66.44 (11.67) | 76.99 (16.87) | 67.07 (14.86) | 4.73** |
| Adaptive Composite | 64.18 (14.15) | 76.14 (14.47) | 68.84 (10.90) | 6.04** |

**Notes:** *p<.05; **p<.01, ***p<.001. The 14 children who were ADOS-2 positive but clinically negative did not significantly differ in their cognitive and adaptive standard scores as the PWS+ASD group. Both of these groups scored lower than their counterparts with PWS only. In the only exception to this pattern, the ADOS-2 positive, clinically negative children had significantly lower VABS-2 Communication domains scores than *both* groups.

**Additional Table 2.** N’s and formulas used to calculate percent positive agreement (PPA) and percent negative agreement (PNA) between the SCQ and ADOS-2.

|  | **ADOS-2 Positive**  **N** | **ADOS-2 Negative**  **N** |
| --- | --- | --- |
| SCQ Positive | (a) 20 | (b) 26 |
| SCQ Negative | (c) 14 | (d) 86 |
| PPA= a/(a+c) x100 | 58.82% (41.89-74.31) |  |
| PNA= d/(b+d) x100 |  | 76.78% (68.30-83.90) |
